# Characterization of the Kidney Transcriptome of the Long-Haired Mouse *Abrothrix hirta* (Rodentia, Sigmodontinae) and Comparison with That of the Olive Mouse *A*. *olivacea*

**DOI:** 10.1371/journal.pone.0121148

**Published:** 2015-04-10

**Authors:** Lourdes Valdez, Facundo Giorello, Matías Feijoo, Juan C. Opazo, Enrique P. Lessa, Daniel E. Naya, Guillermo D’Elía

**Affiliations:** 1 Instituto de Ciencias Ambientales y Evolutivas, Facultad de Ciencias, Universidad Austral de Chile, Valdivia, Chile; 2 Departamento de Ecología y Evolución, Facultad de Ciencias, Universidad de la República, Montevideo, Uruguay; Universtiy of Maryland Schoool of Medicine, UNITED STATES

## Abstract

To understand how small mammals cope with the challenge of water homeostasis is a matter of interest for ecologists and evolutionary biologists. Here we take a step towards the understanding of the transcriptomic functional response of kidney using as a model the long–haired mouse (*Abrothrix hirta*) a species that distributes across Patagonian steppes and Austral temperate rainforests in Argentina and Chile. Specifically, we i) characterize the renal transcriptome of *A*. *hirta*, and ii) compare it with that—already available—of the co-generic and co-distributed *A*. *olivacea*. Renal mRNA transcripts from 16 specimens of *A*. *hirta* from natural populations were analyzed. Over 500 million Illumina paired-end reads were assembled *de novo* under two approaches, an individual assembly for each specimen, and a single *in-silico* normalized joint assembly including all reads from all specimens. The total number of annotated genes was similar with both strategies: an average of 14,956 in individual assemblies and 14,410 in the joint assembly. Overall, 15,463 distinct genes express in the kidney of *A*. *hirta*. Transcriptomes of *A*. *hirta* and *A*. *olivacea* were similar in terms of gene abundance and composition: 95.6% of the genes of *A*. *hirta* were also found in *A*. *olivacea* making their functional profiles also similar. However, differences in the transcriptome of these two species were observed in the set of highly expressed genes, in terms of private genes for each species and the functional profiles of highly expressed genes. As part of the novel transcriptome characterization, we provide distinct gene lists with their functional annotation that would constitute the basis for further research on these or any other species of the subfamily Sigmodontinae, which includes about 400 living species distributed from Tierra del Fuego to southern United States.

## Introduction

During the last decade, interdisciplinary integration and accelerated technological advances have expanded the horizon of Evolutionary Biology [1], [2]. Ecological and Evolutionary Genomics incorporates a functional approach to gain insight into the genetic basis of ecologically important traits [3] and to the understanding of the forces and mechanisms that drive evolutionary change [4], [5], [6]. Beyond providing molecular information that is useful for designing genome-wide markers for phylogeographic, population genetics and phylogenomics studies (e.g., [7], [8], [9]), contributions are shedding light on neutral and adaptive processes in natural populations [10], mechanisms of adaptation, speciation and hybridization (see [4] and references therein). Stepping towards the understanding of physiological function in natural conditions, here we address renal gene expression in individuals of the South American long-haired mouse, *Abrothrix hirta* [11], collected in natural populations. This sigmodontine rodent is widely distributed in southern South America; it occurs along both sides of the Andes, from about the Chilean Region of Maule (ca. 35° S) and the Argentinean province of Mendoza (ca. 33° S) southwards to Tierra del Fuego (ca. 52° S) [12], [13]. This work has two main goals. First, we characterize the renal transcriptome of *A*. *hirta* via RNA-seq based on specimens collected in natural populations in forests and steppes from southern Argentina and Chile. Second, we compare the renal transcriptome of *A*. *hirta* with the recently described renal transcriptome of the co-generic and co-distributed olive field mouse *A*. *olivacea* [14]. These two species are sympatric through most of their distributional ranges [12], [13] and were simultaneously collected at the same geographic localities. The large amount of molecular information generated will be useful for further studies on sigmodontine species because available genomic resources for this group are scarce; e.g., only two completely sequenced genomes of other members of the family Cricetidae, none of them a sigmodontine, are available in GenBank, and, to the best of our knowledge, only a single transcriptome of a sigmodontine rodent has been studied to date [14].

## Materials and Methods

### Specimens sampling and ethic statements

Sixteen adult specimens (8 males, 8 females) of *Abrothrix hirta*, a non-endangered species, were included in this study. Animals were collected from natural populations in Gan Gan (Chubut), and Río Oro (Santa Cruz) in Argentina, and in San Martín and Chabelita (Los Ríos) and Sector Barrancoso (Aysén) in Chile. Geographic coordinates for each locality, together with biological information for each specimen are given in Table A in S1 File. No specific permission was required to work at these localities. Annual precipitation widely varies in the study area, ranging from 170 to 2,200 mm [15]. Animals were captured using Sherman live traps baited with oatmeal. Traps were activated during the night and captured animals were removed early in the morning. All field trips were conducted during the fall season (April 2011 and March 2012).

Immediately after sacrifice by cervical dislocation, the right kidney was removed and frozen in liquid nitrogen; all samples were later stored at -80°C until laboratory procedures were performed. Field identification of specimens was based on morphological features followed by posterior corroboration-with analyses of *cytochrome-b* gene sequences as in [16]. Specimen vouchers were deposited in the Colección de Mamíferos de la Universidad Austral de Chile, Valdivia, Chile and Colección de Mamíferos del Centro Nacional Patagónico, Puerto Madryn, Argentina. All procedures involving live animals were conducted following guidelines of the American Society of Mammalogists [17] and were approved by the Uso de Animales en la Investigación committee of the Universidad Austral de Chile (permit 04/11).

### Laboratory procedures

One-half of the right kidney of each individual (after sagittal section) was subjected to RNA extraction, library construction and sequencing. Total RNA was extracted from each kidney using the RNeasy mini kit (Qiagen) that produces a mRNA-enriched purification. Quality and concentration of RNA products were determined by UV-absorbance spectrophotometry (Nanodrop technologies inc. Wilmington, DE, USA). A value higher than 1.8 for absorbance ratios 260/280 and 260/230 indicated a good quality of purification products relative to protein and inorganic contaminants, respectively. Integrity of RNA molecules was checked with formaldehyde-agarose denaturing gels and using a 2100 Bioanalizer platform (Agilent Technologies) with RNA Integrity Numbers (RINs) of 8 or higher. Purified total RNA samples were shipped to Macrogen Inc. (Seoul, South Korea) under recommended RNA submission conditions. Messenger-RNA (mRNA) content was purified from total RNA with the PolyATract mRNA Isolation System II (Promega Inc.) and copied to cDNA molecules using Illumina TruSeq RNA Sample Preparation Kit v2. One such cDNA library was constructed for each specimen; libraries were subjected to massive sequencing following Illumina HiSeq2000 protocol (Illumina Inc. www.illumina.com [18]).

### Bioinformatic analysis

#### De novo assembly, gene and functional annotation

Primary analyses of the reads were conducted with Illumina Pipeline (CASAVA) v1.8.2, including the removal of adapter sequences. Quality of reads was examined using FastQC (Babraham Institute, http://www.bioinformatics.bbsrc.ac.uk/projects/fastqc) and reads files were depurated from bases with Q values [19] lower than 24—which corresponds to an error rate lower than 1% (but see [20], [21] where it is suggested that this threshold may be unnecessarily high)—using the FastX-Toolkit trimmer tool version 0.0.13.2 [22]. Trimmed reads were deposited at the National Center for Biotechnology Information (NCBI) Sequence Read Archive (SRA) under SRA study accession number SRP044911 (http://www.ncbi.nlm.nih.gov/sra/?term=SRP044911; Table 1).

**Table 1:** RNA-seqdata and results of *de novo* assembly and gene annotation of 16 renal transcriptomes of *Abrothrix hirta*.

Library	Read count (read pairs)	Read length^1^	Assembled contigs	Contigs with significant hits^2^	Identified genes	SRA accession	TSA accession
GD1434	28,743,071	94	53,486	26,651 (49.8)	11,538	SRR1531531	GCIC01000000
GD1452	33,871,900	84	71,133	41,559 (58.4)	12,716	SRR1531537	GCJK01000000
GD1454	33,246,494	74	64,161	35,434 (55.2)	12,029	SRR1531538	GCHF01000000
GD1455	37,596,078	74	56,835	33,603 (59.1)	11,598	SRR1531539	GCHG01000000
GD1493	37,628,054	74	72,737	44,194 (60.8)	12,126	SRR1531540	GCHH01000000
GD1494	32,682,662	74	54,950	32,142 (58.5)	11,107	SRR1531541	GCJI01000000
GD1514	35,379,784	69	46,472	24,199 (52.1)	10,968	SRR1531522	GCIR01000000
GD1529	30,368,779	94	77,723	33,091 (42.6)	13,197	SRR1531532	GCIS01000000
PPA251	30,565,533	89	69,639	38,173 (54.8)	12,646	SRR1531533	GCHK01000000
PPA252	27,317,385	49	35,810	21,689 (60.6)	9,094	SRR1531534	GCHI01000000
PPA305	33,342,460	84	71,723	33,195 (46.3)	12,996	SRR1531535	GCJH01000000
PPA326	28,764,859	49	38,448	22,450 (58.4)	9,972	SRR1531536	GCJD01000000
PPA357	39,539,322	79	70,082	37,409 (53.4)	12,184	SRR1538450	GCJE01000000
PPA440	40,432,573	79	76,357	37,818 (49.5)	12,779	SRR1531542	GCHN01000000
PPA527	38,265,717	74	65,982	35,526 (53.8)	12,211	SRR1531543	GCHL01000000
PPA528	38,251,645	84	76,018	41,122 (54.1)	12,590	SRR1538451	GCHM0100000
**Mean**	**34,124,770**	**76.5**	**62,597**	**33,640(54.2)**	**11,859**		
**Total**	**545,996,316**	–	**1,001,556**	**538,255**	**14,956** ^*^		

Libraries are identified with specimen number.

^1^Length after quality trimming

^2^e-value < 1e-10.

*Distinct genes in the individual assembly strategy

We implemented two assembly approaches. First, reads from each specimen were assembled independently using Trinity (trinityrnaseq-r2013-02-15 [23]) using default settings except for—group_pairs_distance, that was adjusted according to contig lengths reported in individual NGS library reports; see Table A in S1 File). As a second approach, we conducted a joint assembly of the reads from all 16 specimens. To reduce the usage of computational resources, this dataset was submitted to digital normalization prior to the assembly using Trinity *in-silico* read normalization [24] (Trinity—normalize_reads <other assembly parameters as above >). This strategy has proven effective for transcriptome characterization in a closely related species [14]. Transcriptome Shotgun Assembly projects have been deposited at DDBJ/EMBL/GenBank under bioproject PRJNA256304.

Gene annotation for the resulting assembled contigs from both strategies was determined on the basis of significant alignments (e-value < 1e-10) against protein isoforms of *Mus musculus* available at OMA Browser [25]. Searches were performed using the program BLASTx in its parallel implementation mpiBLAST version 1.6.0 [26]. BLASTx best hits were recorded using MOUSE protein IDs (i.e., the label for *Mus musculus* in OMA project); then these IDs were grouped under corresponding Ensembl gene IDs for *Mus musculus* (ENSMUSG) for functional analyses using DAVID Bioinformatics Resources 6.7 [27], [28]. DAVID was also used to assess the functional categories that are significantly overrepresented in a particular group of genes relative to the totality of renal genes identified in *A*. *hirta*.

In order to compare the performance of each assembly approach, the outcome of each assembly group (i.e., joint and individual assemblies) was examined for the proportion of annotated contigs that reconstruct the length of coding sequences (CDS) of *Mus musculus* in at least 50, 70, 80 and 90%. Estimations were based on significant hits on BLASTx searches (e-value 1e-10) at each cut-off % relative to the total number of annotated contigs.

#### Gene expression analysis and comparison of the transcriptome of *A*. *hirta* and *A*. *olivacea*

Gene expression level was estimated on the basis of read count. First, reads from individual libraries were aligned, in a paired-end fashion, to their own set of annotated contigs using the alignment program Bowtie, that is implemented in RSEM v1.2.5 [29]. Then, values of gene-level transcript per million (TPM) were calculated using RSEM. Following Giorello et al. [30], average TPM per gene were used to generate a consensus gene expression ranking, and the top 5% of this ranking were taken into account for further comparison of highly-expressed genes in the kidney of *A*. *hirta* relative to those of the co-generic *A*. *olivacea* [14]. We cross-referenced the lists of highly expressed genes (top 5%) in each species to assess consistency across species.

Similarity in the pattern of renal gene expression among individuals of *A*. *hirta* and *A*. *olivacea* was examined by means of multidimensional scaling (MDS). Analyses were conducted on two datasets, one including all genes recovered in all specimens of both species and another restricted to the top 5% of highly expressed genes.

## Results

### Read assembly and gene annotation in *A*. *hirta*

The renal transcriptome of sixteen individuals of *Abrothrix hirta* was sequenced from cDNA libraries with a range of modal length between 152 and 385 bp. Massive sequencing of each library yielded over 130 million 101-bp-long Illumina paired-end reads. Reads were 49–94 bp long after excision at the 3’ end of bases with a quality Q-value below 24 (Table 1). A total of ~83.8 Gbp in over 500 million reads were used for bioinformatics analyses.

Seventeen independent assemblies were generated; a normalized joint assembly including all 16 transcriptomes of *Abrothrix hirta* and one individual assembly for each one of the 16 transcriptomes. In the former, *in-silico* normalization reduced the original number of reads from 545,996,316 to 25,787,195 pairs, which is less than the mean read count in individual libraries (Table 1).

Individual assemblies generated, on average, 62,597 contigs, whereas the joint assembly yielded almost five times more contigs, 294,787 (Tables 1 and 2). Despite this difference, gene annotation based on BLASTx searches against OMA mouse protein database resulted in similar proportions of annotated contigs in both assembly strategies, i.e., 54.2% on average for individual assemblies and 57.3% for joint assembly (Table 1).

**Table 2:** Main assembly metrics for the individual and joint assembly of 16 renal transcriptomes of *Abrothrix hirta*.

	range of individual libraries		Joint assembly
	minimum	maximum	
total reads	54,634,770	80,865,146	1,091,992,632
Reads after normalization	–	–	25,787,195
read length	49	94	49–94
total contigs	21,689	44,194	294,787
total contigs with significant hits (%)	21,689 (60.6)	44,194 (60.8)	168,979 (57.3)
max contig length	7,288	18,234	17,645
min contig length	201	201	201
average length	693	1,301	1,839
median length	349	573	1,276
total genes	9,094	13,197	14,410

Gene annotation of the 16 individually assembled transcriptomes led to the identification of 14,956 distinct genes in the kidney of *A*. *hirta*. About 51% of these genes (7,582 genes) were found in all 16 specimens studied. On average, 11,859 genes (9,094–13,197; Table 1) were identified in each individual.

**Fig 1:**
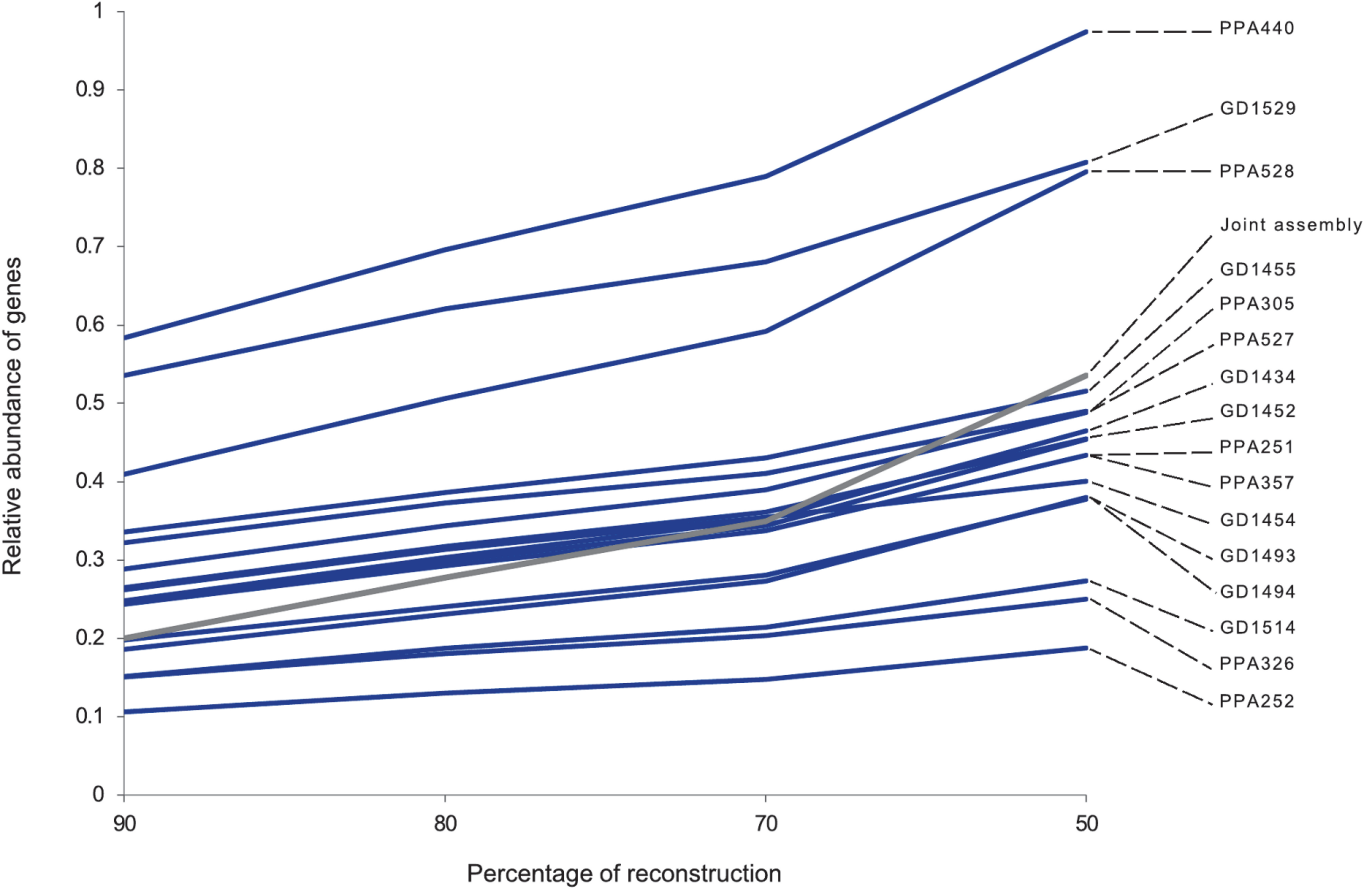
CDS reconstruction levels for 16 individual assemblies and one joint assembly of renal transcriptomes of *Abrothrix hirta*. The graph shows the proportion of contigs that contains 90, 80, 70 and 50% of the length of the correspondent gene of *Mus musculus*.

**Fig 2:**
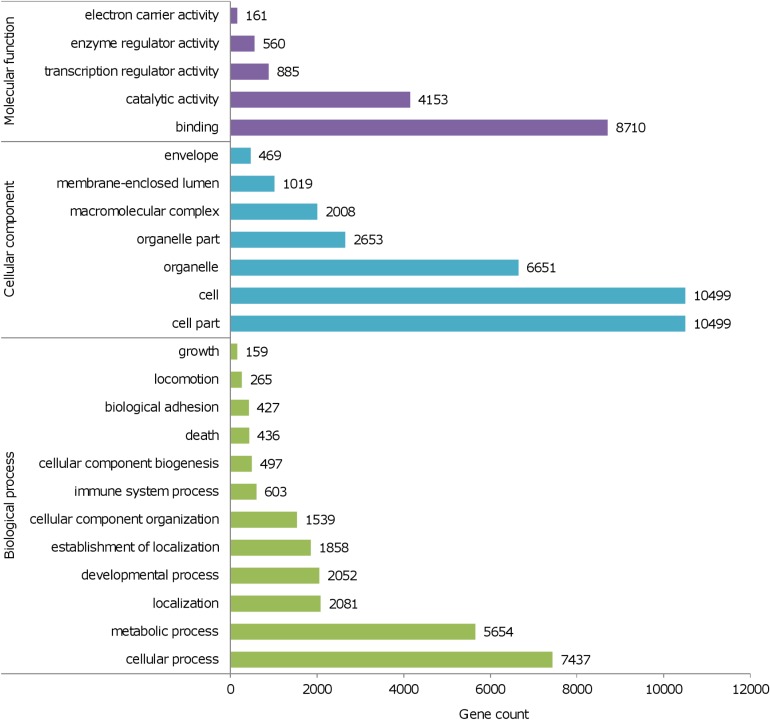
Functional annotation for the expressed renal genes of *Abrothrix hirta* reconstructed in a joint assembly of 16 individual transcriptomes. Gene abundance at the higher level terms of Gene Ontology, using DAVID [27] tools and databases, are shown for biological process (BP_FAT), cellular component (CC_FAT) and molecular function (MF_FAT).

Compared to the individual assembly approach, the joint assembly led to a similar number of annotated genes, i.e., 14,410 (Table B in S1 File). About 97% of these genes (13,903 out of 14,410 genes) were also recovered in at least one individual transcriptome. The set of annotated genes from the joint assembly also contains 94.5% of those genes that were found in all 16 individual transcriptomes (i.e., out of the 7,582 genes common to all specimens, 7,390 were also annotated in the joint assembly). Regarding the proportion of reconstruction of mouse CDS, the performance of the joint assembly was comparable to most individual assemblies (Fig 1). Higher-level functional annotation of the 14,410 genes recovered in the joint assembly showed that most genes in the renal transcriptome of *A*. *hirta* participate in cellular and metabolic processes (e.g., regulation of transcription and regulation of RNA metabolic process; Fig 2; Table C in S1 File).

Taking into account the results of gene annotation of joint as well as individual assemblies, a total of 15,463 distinct genes were identified in the renal transcriptome of *A*. *hirta*. Both strategies identified 13,903 genes in common (i.e., 96% of the gene list from the joint assembly and 93% of the list of distinct genes from the summation of individual assemblies). About 3% (507 genes out of 14,410) of the genes annotated in the joint assembly were not found in any individual transcriptome. Conversely, 1,053 genes that were identified in at least one individual transcriptome were not found with the joint strategy. The list of these 1,053 genes is provided in the Table D in S1 File, and its functional characterization in Table E in S1 File.

### Highly expressed genes in the kidney of *A*. *hirta*

Lists of the top 5% more highly expressed genes, obtained separately from each individual transcriptome, contain on average 593 genes (range: 455 to 660 genes). A total of 183 genes were common to the top 5% most expressed genes list of all renal transcriptomes (Tables V and W in S1 File; see Tables B to U for gene expression levels in individual transcriptomes); for purposes of interspecific comparisons (see below) these 183 genes were taken as a consensus list of highly expressed genes for the renal transcriptome of *A*. *hirta*. This top 5% most-expressed gene set of the kidney transcriptome of *A*. *hirta* was significantly enriched, at the most inclusive level, in GO terms related to cellular processes (e.g., translation), energy metabolism (e.g., oxidative phosphorylation, energy derivation by oxidation of organic compounds), and purine metabolic processes (Tables X and Y in S1 File).

The ontology analysis of highly-expressed genes revealed that the majority of them participate in cellular and metabolic processes (Table X in S1 File). Accordingly, top biochemical pathways in activity are those responsible for the catabolism of amino acids (mostly Arginine, Histidine, Glutamate, Glutamine and Proline) and sugar metabolism (e.g., glycolysis and gluconeogenesis). As it is the case of the top 5% most-expressed genes, functional profile of the joint assembly indicates that most of these genes participate in a wide range of cellular processes, particularly related to cellular metabolism (Fig 2; Table C in S1 File). At the molecular level, catalytic activity is the most abundant function in both profiles; accordingly, cell and organelle components are the most abundant terms (Fig 2; S1 Fig).

A multidimensional scaling analysis showed that specimens of *A*. *hirta* do not segregate according to sex in relation to transcriptome structure; this was observed when considering expression levels of 6,303 common genes in all individual transcriptomes (S2A Fig), as well as when restricting the analysis to top 5% most-expressed genes (S2B Fig).

### Comparison of the renal transcriptomes of *A*. *hirta* and *A*. *olivacea*

Transcriptome composition in the kidney transcriptome of *A*. *hirta*, as revealed by the gene annotation of the joint assembly, exhibited a similarity of 95.6% with that of *A*. *olivacea* (i.e., 13,782 genes in common). Therefore, functional profiles are similar in both species (Fig 2). A thorough functional categorization for the 14,410 genes found in the renal transcriptome of *A*. *hirta* contains 859 higher-level GO terms (Table C in S1 File).

Comparison of the consensus top 5% highest-expressed genes for *A*. *hirta* and *A*. *olivacea* revealed 140 genes in common; i.e., 76.5% of the highest-expressed genes in *A*. *hirta* (140 genes out of 183) were also found in high expression in the kidney of *A*. *olivacea* (Table V in S1 File). Most of these common genes encode proteins located in non-membrane-bounded organelles and are involved in metabolic processes (Table X in S1 File). The functional profile at the most general level for this gene group (S1 Fig) shows that most represented functional categories are the same that those in the profile of all genes (Fig 2).

Functional characterization of the genes in the top 5% of *A*. *hirta* and *A*. *olivacea* share very few GO terms (Tables X, Y and Z in S1 File). All 43 genes that were found uniquely in *A*. *hirta* at the 5% cutoff were found to be ranked within the top 22% highest-expressed genes in *A*. *olivacea* (Table AA in S1 File); meanwhile, the 143 genes unique to *A*. *olivacea* at the 5% cutoff were found to be ranked among the top 12% in *A*. *hirta* (Tables AB and AC in S1 File).

A multidimensional scaling analysis conducted with the expression levels of the 6,303 genes that were found in all specimens of in *A*. *hirta* and *A*. *olivacea* revealed that individuals do not group per species (S3A Fig). Although without a clear diagnostic value, a MDS restricted to common highly-expressed genes shows a tendency of co-specific specimens to group in the multidimensional space (S3B Fig).

## Discussion

### Assembly strategy for transcriptome characterization

Characterizing the kidney transcriptome of *A*. *hirta* based on RNA-seq data from 16 individuals posed the challenge of effectively compiling biological variation contained in a dataset of more than 500 million read pairs. The strategy adopted to face this challenge, i.e., the joint assembly with digital normalization of reads, succeeded in reducing read count to a value near the average size of individual libraries (Tables 1 and 2). At the same time, this strategy accounted for the variation contained in all specimens into a set of contigs that is five times larger than the average obtained from the analysis of individual assemblies and four times smaller than the sum of contigs from the 16 libraries (Table 1). Gene annotation of the joint assembly led to the identification of a number of genes that is similar to that found at individual assemblies (Tables 1 and 2); 97% of the genes in the joint assembly were recovered in both strategies. The remaining 3% is composed of 507 genes that were not found in any individual transcriptome. This set might include low-expression genes and/or spurious assembly reconstructions.

The sum of individual assemblies recovered 1,053 genes more than the normalized joint assembly; of these genes, 546 were present in all individual assemblies (Tables D and E in S1 File). It is noteworthy that the analysis of the renal transcriptome of *A*. *olivacea* exhibited an opposite pattern, i.e., the number of annotated genes that resulted from the normalized joint assembly was higher than that derived from the sum of individual assemblies [14]. Future studies would clarify which of these patterns is more frequent, as well as the underlying causes and factors that affect them.

Another issue worthy of consideration when implementing a joint assembly is the integrity of reconstructed genes. Any particular gene sequence (or set of gene sequences) might be more completely represented in a particular individual assembly than it would be in the joint assembly. In the latter, the proportion of reconstructed CDS above 80% of its length was lower than that from most individual assemblies (Fig 1; see [14] for a discussion on normalized vs. non-normalized strategies). Therefore, this strategy may not be the best choice when sequence completeness is a priority. Yet, our overall results support the normalized joint assembly strategy as an effective approach to transcriptome characterization while taking into account the biological diversity found in a high number of individuals.

### Gene expression in the kidney of *A*. *hirta*

Assuming that the genome of *A*. *hirta* contains about the same number of protein-coding genes documented in *Mus musculus* (22,011–22,444; [30]), our results suggest that approximately 64–68% of the total protein coding genes are expressed in the kidney of *A*. *hirta* (Table 2). The number of genes expressed in the kidney and sequenced via transcriptome analysis for the baboon [31] and three heteromyid rodents [3], [32] are similar to our reconstructions.

Of the total genes annotated in the species transcriptome, at least 80% were found to be expressed in the kidney of any particular individual of *A*. *hirta* (Tables 1 and 2). That is, on average, 11,895 out of 14,410 genes (in the most conservative estimation; Table B in S1 File) were found to be expressed in a particular individual. Noteworthily, although the number of expressed genes is similar in all individuals, gene composition among them is highly variable; only about 51% of the total annotated kidney genes were found in expression in the transcriptome of all individuals (i.e., out of the total of 14,956 distinct genes annotated in the individual assembly approach, 7,582 genes were found in all specimens). The proportion of common genes found in all libraries of *A*. *hirta* was in line with that found by Giorello et al. ([14]; see also references therein) where 66% of the renal genes were found in all 12 individual transcriptomes analyzed.

Moreover, individual variation was even greater for the 5% highest expressed genes in the renal transcriptome of *A*. *hirta*: only about 30% of the genes in this group were present in the 16 individual transcriptomes (i.e., 183 genes out of an average of 583 genes; Tables V and W in S1 File). Given that the top 5% list was built based on gene abundance, the magnitude of the variation can be attributed to differences in gene expression levels. This means that expression levels of about 70% of the highly expressed genes in a given individual is lower than the 5% cutoff, or even that those genes are not expressed at all, in at least one of the other 15 analyzed individuals.

Several points should be considered when discussing interindividual variation. From a technical standpoint, sequencing depth cannot be ruled out as affecting the observed gene composition; even when our representation of each individual transcriptome (20–40 million read pairs) is in line with the recommended read count for non-model animals [33]. Regarding experimental design, the study of individuals from natural populations, in particular of small mammal species, has its own limitations. One of them concerns the time that animals spent in the trap before they were discovered and removed; certainly, there would always be differences among individuals in these time periods, and consequently, some animals would be more stressed (e.g., dehydrated) than others. As we were interested in gene expression levels in natural conditions (i.e., wild genomics), the just mentioned putative effect of sampling time could not be removed through acclimation of specimens to controlled conditions. Importantly, we note that the observed interindividual variation in transcriptome structure is not accounted by differences between sexes; i.e., the transcriptome of male individuals are as different among then as they are from those of female, and vice versa (S2 Fig). Further studies will be required to properly dissect intrinsic biological from methodological factors underlying the observed variation in the individual transcriptomic repertoire.

The group of highly expressed genes in the kidney of *A*. *hirta* contains genes of interest due to their function in osmoregulation. For instance, the water channel Aquaporin 1 plays a role in urine concentration [34], the FXYD domain-containing an ion transport regulator that ensures the correct function of renal sodium reabsorption [35], and the kallikrein 1 (ENSMUSG00000063903) is involved in the regulation of ion transport [36]. Although without a direct relation with osmoregulation, other highly expressed genes, such as the secreted phosphoprotein (ENSMUSG00000029304) and the Cadherin 16 (ENSMUSG00000031881), were also found highly expressed in the kidney transcriptome of the kangaroo rat *Dipodomys spectabilis* [32].

### Comparison of renal transcriptome profiles of *A*. *hirta* and *A*. *olivacea*

Comparative characterization of the kidney transcriptome of *A*. *hirta* reported in this study and that of *A*. *olivacea* reported by Giorello et al. [14] revealed several points of similarity in their general expression profile. First, the total number of reconstructed genes, considering the various approaches implemented, was similar in both species; only 667 additional genes were reconstructed in *A*. *olivacea* (Table 2; [14]). Second, gene composition in both transcriptomes was also similar; 91.4% and 95.6% of the genes found to be expressed in *A*. *olivacea* and *A*. *hirta*, respectively, were common to both species. As a consequence of these two variables, i.e., gene richness and gene composition, the functional characterization of the renal transcriptome of both species is highly congruent (Fig 2; Table C in S1 File). Another point of similarity between the renal transcriptomes of *A*. *hirta* and *A*. *olivacea* emerges when focus is placed in the most-expressed genes. In general, highly expressed genes in one species are also in relatively high expression in the other species (Tables AA and AB in S1 File). For instance, two-thirds of the highly expressed (top 5%) genes of *A*. *hirta* were found to be in the same range in the transcriptome of *A*. *olivacea* (Table V in S1 File), whereas the remaining one-third ranked above 22% in the expression rank of *A*. *olivacea* (Table AA in S1 File).

The analysis of the top 5% genes also revealed the most conspicuous differences between the renal transcriptome of the two rodent species. Highly expressed genes that are unique to each species show markedly dissimilar functional profiles (Tables W, Y, Z and AC in S1 File). For instance, the gene metallothionein 2 (ENSMUSG00000031762), which in the kidney of rodents is associated to metal detoxification [37], was found in high expression in *A*. *hirta* whereas in *A*. *olivacea* its expression was not detected; this opens a window to the exploration of diet-related gene expression patterns. Noteworthy are the results of the MDS showing that individual transcriptomes tend to segregate into species groups when the analysis is restricted to the most expressed genes (i.e., those genes within the top 5%) rather than when all reconstructed genes are considered (S3 Fig). This indicates that species differ more in the expression level of the top 5% expressed genes than they do in their whole transcriptomes. It is interesting to note that this pattern holds even when samples of both species include specimens collected in forest and steppes, habitats that differ by an order of magnitude in precipitation and consequently in productivity (see [15]). Future studies would clarify the consistency of this observation, as well as its causal factors. In this regard, an avenue worth pursuing is the assessment of the influence of distinct diet composition ([38] see also [15]), a factor that also affects water homeostasis [39], [40], and thus, possibly, also the composition of kidney transcriptomes. Likewise, considering that *A*. *olivacea* and *A*. *hirta* are closely related, but not sister species [41], [42]; future characterization of additional sigmodontine renal transcriptomes would clarify the extent to which the high similarity found in the renal transcriptomes of *A*. *olivacea* and *A*. *hirta* is due to history and/or function.

## Concluding remarks

Transcriptome analysis represents a good tool for question-driven exploratory research [43]. Here we have characterized the structure of the renal transcriptome of a South American species of sigmodontine mouse and compared it with the already available transcriptome of a co-generic species. Overall, the joint assembly showed to be a good approach for transcriptome characterization. This approach makes computational data analysis more affordable and allows considering the biological variation present in many individuals. On this particular, results also showed the relevance of incorporating individual variation in order to obtain a more accurate profile of the gene expression of an organ and of a given species.

As far as we know, this contribution and that of Giorello et al. [14] are the first transcriptome characterizations for the large subfamily Sigmodontinae, which include about 400 living species distributed from Tierra del Fuego in southernmost South America to southern United States. Here we provide several gene lists (e.g., genes that are common to both species, highly-expressed genes, as well as their functional categorization) for future studies. We expect this information to be the basis of upcoming ecological genomic studies, and also to be useful for developing molecular markers for population genomics and phylogenomic studies of this diverse group of rodents. As stated in Lessa et al. [44], evolutionary and ecological studies of South American rodents are transitioning into the genomic era and our study and that of Giorello et al. [14] are pioneering this shift for studies of sigmodontines.

## Supporting Information

S1 Fig Functional annotation of the common genes found in the lists of top 5% most-expressed genes in renal transcriptomes of *Abrothrix hirta* and *A*. *olivacea*. Gene abundance for Gene Ontology terms at the higher level, using DAVID [31] tools and databases, are shown for biological process (BP_FAT), cellular component (CC_FAT) and molecular function (MF_FAT).
(TIF)

S2 Fig MDS of 16 renal transcriptomes of male (blue labels) and female (green labels) of *Abrothrix hirta*. Analyses were based on (A) expression levels (TPM) of 6,303 genes and (B) expression levels of 140 top 5% most-expressed genes.
(TIF)

S3 Fig MDS of 16 renal transcriptomes of *Abrothrix hirta* (blue dots) and 13 transcriptomes of *A*. *olivacea* (white dots). Analyses were based on (A) expression levels (TPM) of the 6,303 genes found in both species, and (B) expression levels of the 140 genes common to the top 5% most-expressed genes of both species.
(TIF)

S1 File Table A, Studied specimens of *Abrothrix hirta*, collection localities and mRNA library insert length corresponding to individual transcriptomes. Table B, List of the 14,410 reconstructed genes in the renal transcriptomes of *Abrothrix hirta*. Table C, Functional annotation of the 14,410 reconstructed genes of the renal transcriptome of *Abrothrix hirta*. Table D, List of 1,053 genes that were recovered in the gene annotation of individual assemblies but not in the joint assembly in the kidney of *A*. *hirta*. Table E, Functional annotation of the 1,053 genes that were recovered in the gene annotation of individual assemblies but not in the joint assembly in the kidney of *A*. *hirta*. Tables F to U, Expression level of genes annotated in individual assemblies of 16 renal transcriptomes of *Abrothrix hirta* (details in Table A). Table V, List of top 5% most-expressed genes found in renal transcriptome of *Abrothrix hirta* and *A*. *olivacea*. Table W, Top 5% most-expressed genes identified in the renal transcriptome of *Abrothrix hirta* but not in the top 5% list of *A*. *olivacea*. Table X, GO terms significantly overrepresented in the list of common top 5% most-expressed genes in the renal transcriptomes of *Abrothrix hirta* and *A*. *olivacea*. Table Y, GO terms significantly overrepresented in the list of the 43 top 5% most-expressed genes identified in the renal transcriptome of *Abrothrix hirta* but not in the top 5% list of *A*. *olivacea*. Table Z, GO terms significantly overrepresented in the list of the 143 top 5% most-expressed genes identified in renal the transcriptome of *A*. *olivacea* but not in top 5% list of *A*. *hirta*. Table AA, Comparison of the top 5% most-expressed genes identified in the renal transcriptome of *Abrothrix hirta* with the expression ranking of *A*. *olivacea*. For each of the 43 top 5% genes identified only in *A*. *hirta*, the location in the expression ranking of *A*. *olivacea* was recorded. Genes identified with an * were not found in the renal transcriptome of *A*. *olivacea*. Proportion in each column represents the fraction of the total gene set that is included at that specific rank in a given transcriptome (column to the left). Maximum value of average proportion represents the point at the ranking of *A*. *olivacea* that contains all the genes in the list of top 5% most-expressed of *A*. *hirta* (those genes marked with * are not considered). Table AB, Comparison of the top 5% most-expressed genes identified in the renal transcriptome of *Abrothrix olivacea* with the expression ranking of *A*. *hirta*. For each of the 143 top 5% genes identified only in *A*. *olivacea*, the location in the expression ranking of *A*. *hirta* was recorded. Genes identified with an * were not found in the renal transcriptome of *A*. *hirta*. Proportion in each column represents the fraction of the total gene set that is included at that specific rank in a given transcriptome (column to the left). Maximum value of average proportion represents the point at the ranking of *A*. *hirta* that contains all the genes in the list of top 5% most-expressed of *A*. *olivacea* (those genes marked with * are not considered). Table AC, Top 5% most-expressed genes identified in the renal transcriptome of *Abrothrix olivacea* but not in the top 5% list of *A*. *hirta*.
(XLS)
