# Supplementary figures and images for: Characterization of the Kidney Transcriptome of the Long-Haired Mouse Abrothrix hirta (Rodentia, Sigmodontinae) and Comparison with That of the Olive Mouse A. olivacea
Source: PLoS One. 2015 Apr 10;10(4):e0121148. doi: 10.1371/journal.pone.0121148 (PMC4393222; doi:10.1371/journal.pone.0121148)

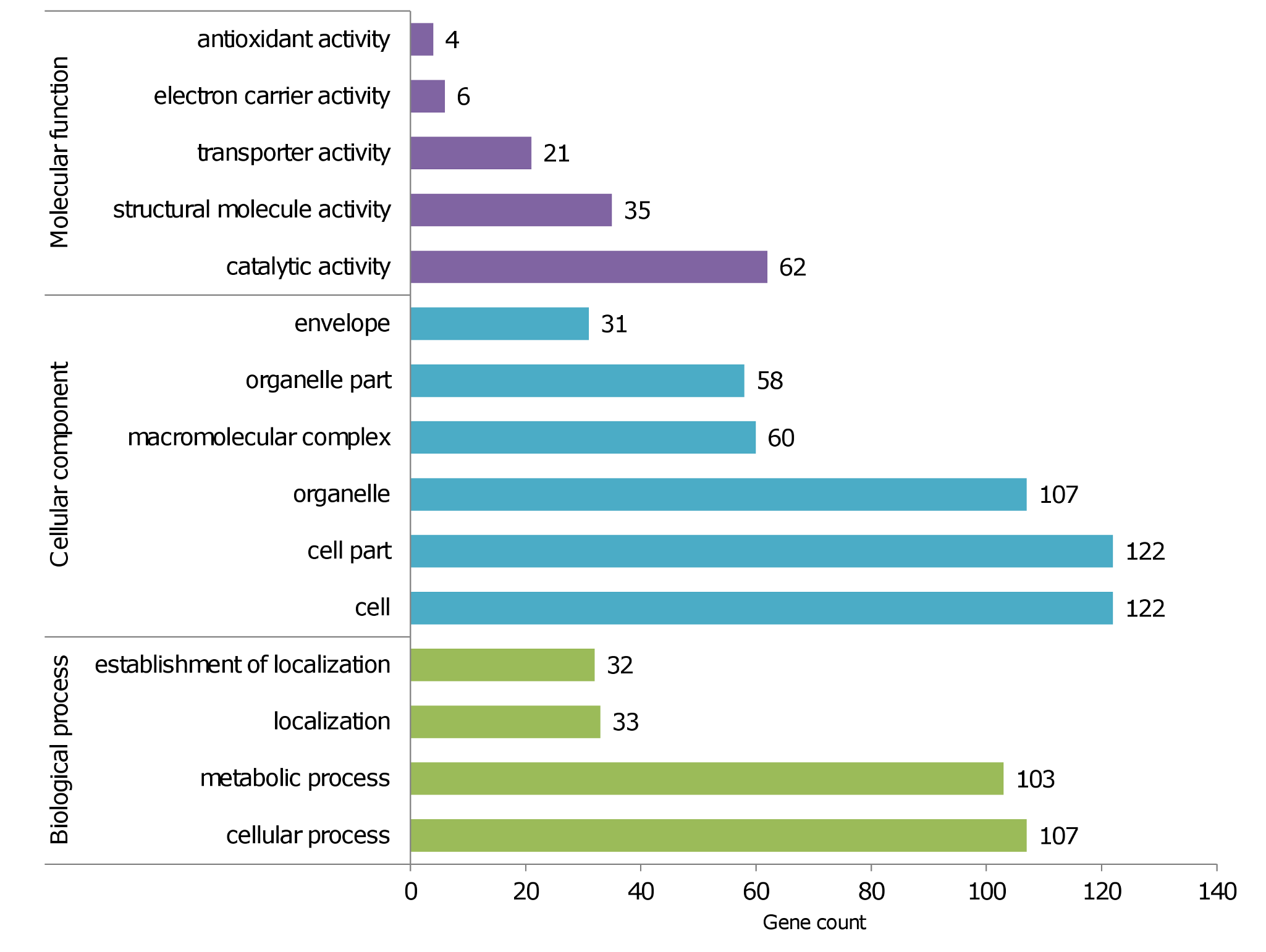

Supplement: S1 Fig — Gene abundance for Gene Ontology terms at the higher level, using DAVID [31] tools and databases, are shown for biological process (BP_FAT), cellular component (CC_FAT) and molecular function (MF_FAT). (TIF) [file pone.0121148.s001.tif]

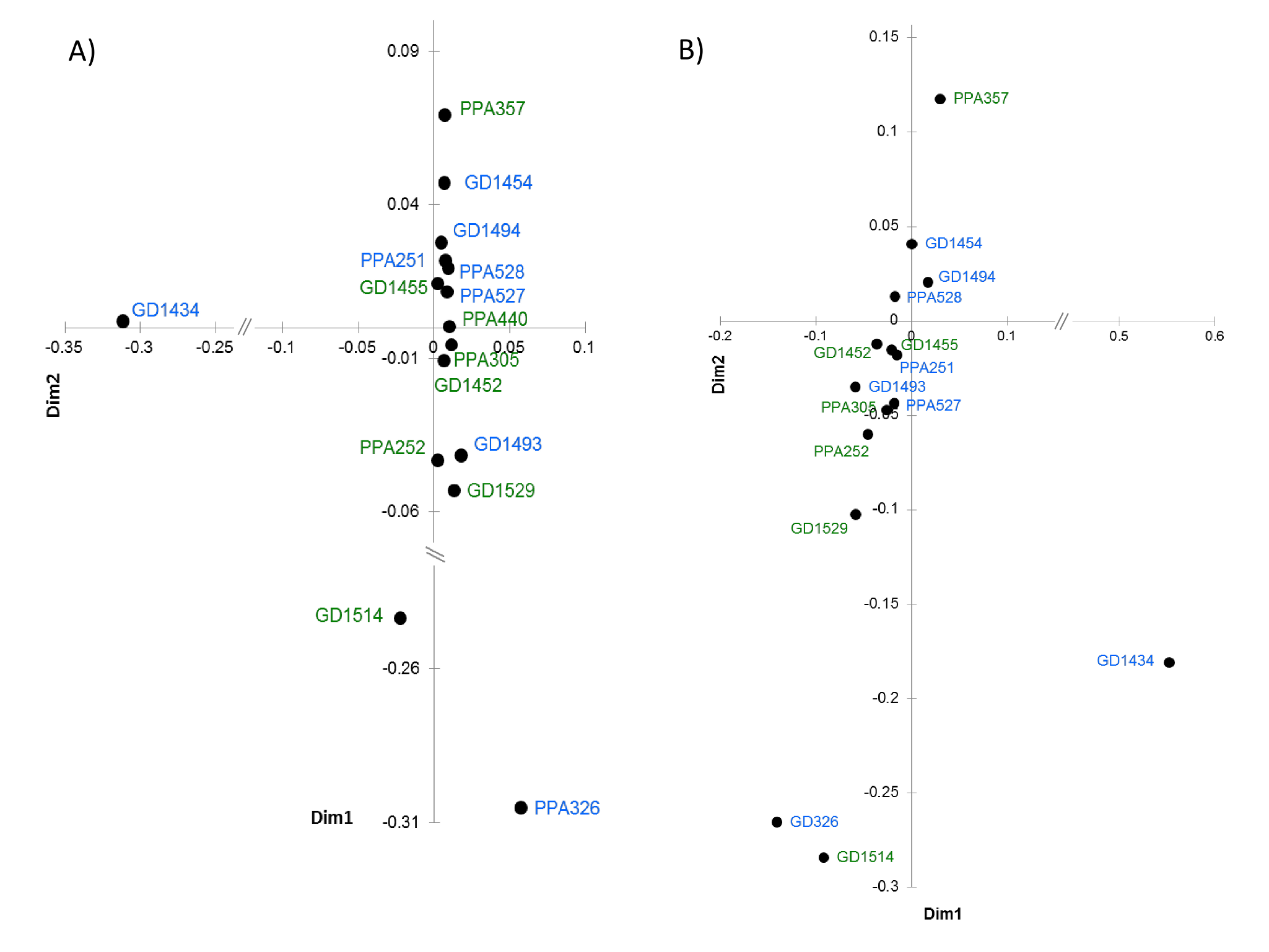

Supplement: S2 Fig — Analyses were based on (A) expression levels (TPM) of 6,303 genes and (B) expression levels of 140 top 5% most-expressed genes. (TIF) [file pone.0121148.s002.tif]

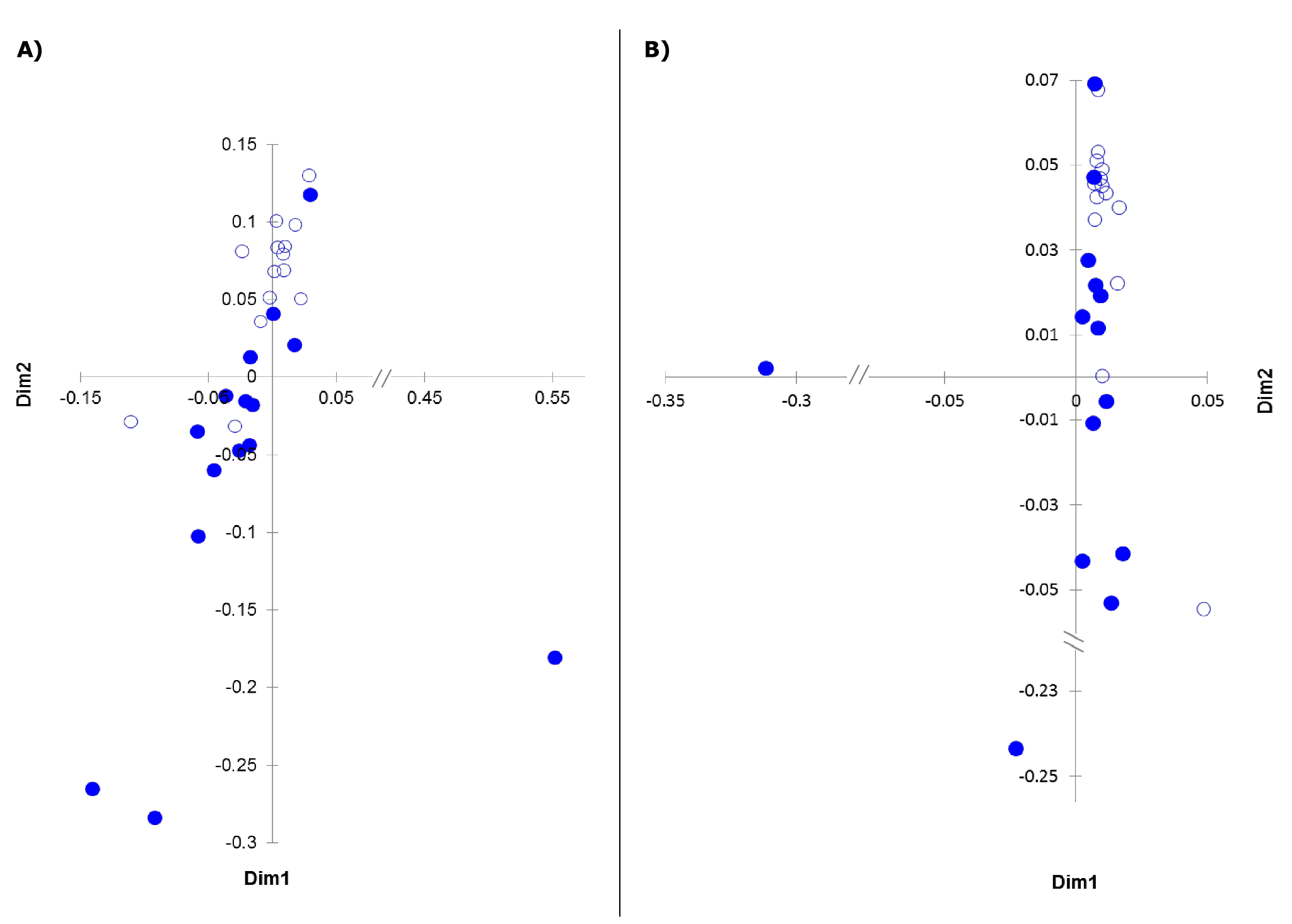

Supplement: S3 Fig — Analyses were based on (A) expression levels (TPM) of the 6,303 genes found in both species, and (B) expression levels of the 140 genes common to the top 5% most-expressed genes of both species. (TIF) [file pone.0121148.s003.tif]
